# Identification of diagnostic gene biomarkers and immune infiltration in patients with diabetic kidney disease using machine learning strategies and bioinformatic analysis

**DOI:** 10.3389/fmed.2022.918657

**Published:** 2022-09-29

**Authors:** Shaojie Fu, Yanli Cheng, Xueyao Wang, Jingda Huang, Sensen Su, Hao Wu, Jinyu Yu, Zhonggao Xu

**Affiliations:** ^1^Department of Nephrology, The First Hospital of Jilin University, Changchun, China; ^2^Department of Urology, The First Hospital of Jilin University, Changchun, China

**Keywords:** diabetic kidney disease, immune infiltration, diagnostic biomarker, bioinformatic analysis, machine learning strategy

## Abstract

**Objective:**

Diabetic kidney disease (DKD) is the leading cause of chronic kidney disease and end-stage renal disease worldwide. Early diagnosis is critical to prevent its progression. The aim of this study was to identify potential diagnostic biomarkers for DKD, illustrate the biological processes related to the biomarkers and investigate the relationship between them and immune cell infiltration.

**Materials and methods:**

Gene expression profiles (GSE30528, GSE96804, and GSE99339) for samples obtained from DKD and controls were downloaded from the Gene Expression Omnibus database as a training set, and the gene expression profiles (GSE47185 and GSE30122) were downloaded as a validation set. Differentially expressed genes (DEGs) were identified using the training set, and functional correlation analyses were performed. The least absolute shrinkage and selection operator (LASSO), support vector machine-recursive feature elimination (SVM-RFE), and random forests (RF) were performed to identify potential diagnostic biomarkers. To evaluate the diagnostic efficacy of these potential biomarkers, receiver operating characteristic (ROC) curves were plotted separately for the training and validation sets, and immunohistochemical (IHC) staining for biomarkers was performed in the DKD and control kidney tissues. In addition, the CIBERSORT, XCELL and TIMER algorithms were employed to assess the infiltration of immune cells in DKD, and the relationships between the biomarkers and infiltrating immune cells were also investigated.

**Results:**

A total of 95 DEGs were identified. Using three machine learning algorithms, *DUSP1* and *PRKAR2B* were identified as potential biomarker genes for the diagnosis of DKD. The diagnostic efficacy of *DUSP1* and *PRKAR2B* was assessed using the areas under the curves in the ROC analysis of the training set (0.945 and 0.932, respectively) and validation set (0.789 and 0.709, respectively). IHC staining suggested that the expression levels of DUSP1 and PRKAR2B were significantly lower in DKD patients compared to normal. Immune cell infiltration analysis showed that B memory cells, gamma delta T cells, macrophages, and neutrophils may be involved in the development of DKD. Furthermore, both of the candidate genes are associated with these immune cell subtypes to varying extents.

**Conclusion:**

*DUSP1* and *PRKAR2B* are potential diagnostic markers of DKD, and they are closely associated with immune cell infiltration.

## Introduction

The increase in the prevalence of diabetes mellitus (1) has had a major impact on the prevalence of diabetic kidney disease (DKD), which is the cause of approximately 50% of cases of end-stage renal disease (ESRD) in developed countries (2); the prevalence of DKD has also been increasing in China (3). Therefore, DKD is now placing a substantial burden on the healthcare system (4). Fibrosis, hypertrophy, and the accumulation of extracellular matrix around glomerular and tubular cells are typical pathological changes in DKD (5). The progression of DKD is associated with microalbuminuria in the early stages and uremia in the later stages (6). Although renal biopsy is the most accurate method of diagnosis, it is not widely used because of its invasiveness. Instead, the diagnostic criteria are typically based on the clinical manifestation of the disease and vary around the world. These criteria include albumin/creatinine ratio, estimated glomerular filtration rate, serum creatinine concentration, the results of renal ultrasonography, and the presence of retinopathy (7). However, these parameters are not highly sensitive or specific diagnostic measures and do not reflect the severity of the renal damage. It is universally accepted that proteinuria is negative in the early stage of DKD and microalbuminuria may revert back to normal urinary albumin excretion rates over declined renal function (8, 9). Therefore, more accurate biomarkers that could be used for the early diagnosis of DKD would facilitate early intervention and improve the outcomes of patients.

Machine learning is increasingly being used to help identify genes that may have diagnostic potential, and the accuracy of identification of genes that are differentially expressed on microarrays has markedly improved (10). With more and more studies revealing the role of immune system and immune cells in the occurrence and development of DKD (11), people gradually regard DKD as an immune-mediated disease (12). Currently, immune infiltrating cells mediated oxidative stress process is considered to play an important role in the occurrence and development of DKD (13). Animal experiments also showed that the number and activity of immune cells in the kidney are related to the renal damage (14). At present, some drugs have been confirmed by experiments that influence the protein expression in renal tissues interacting with infiltrated immune cells could improve DKD fibrosis (15), which provides new therapeutic strategies for the treatment of DKD. Therefore, it is important to reveal the infiltration of immune cells in kidney tissue of DKD and explore the relationship between the identified biomarkers and immune infiltrating cells.

In the present study, we first downloaded microarray datasets obtained from patients with DKD from the Gene Expression Omnibus (GEO) database, merged them, and performed differential expression analysis. Gene set enrichment analysis (GSEA) was then performed using the obtained differentially expressed genes (DEGs), and three different machine learning algorithms were used to identify potential diagnostic markers for DKD. Subsequently, we utilized CIBERSORT, XCELL and TIMER to understand the features of the immune cell infiltration into the kidneys of patients with DKD, in comparison to normal renal tissue. Finally, we assessed the relationships between the identified candidate diagnostic markers and the immune infiltration to further investigate the molecular mechanisms of the progression of DKD.

## Materials and methods

### Identification of differentially expressed genes

Microarray datasets for renal glomeruli from patients with DKD and controls (GSE30528, GSE96804, and GSE99339) were obtained from the GEO database^1^ (16). The three data sets were merged and used as a training set, and the inter-batch differences were corrected for using the SVA package in R (17). The limma package in R was then used for the identification and normalization of the differentially expressed genes (DEGs) by comparing the expression levels in the glomeruli from controls and patients with DKD (18). An adjusted *P*-value < 0.05 and a | log fold change (FC)| > 1 were the criteria used to define DEGs.

### Functional enrichment analysis

Gene Ontology (GO) and Kyoto Encyclopedia of Genes and Genomes (KEGG) enrichment analyses of the enriched DEGs were performed through the “clusterProfiler” package in R, and an adjusted *P*-value < 0.05 was considered to represent statistical significance (19). GSEA was conducted to identify the functional terms that significantly differed between the DKD and control samples (20). We used “c2.cp.kegg.v7.4.symbols.gmt” from the Molecular Signatures Database (MSigDB) as the reference gene set and screened for significantly enriched genes using an adjusted *P*-value of <0.05.

### Screening for potential diagnostic biomarkers

To identify the potential diagnostic biomarkers for DKD, three machine learning algorithms were used to predict disease status: the least absolute shrinkage and selection operator (LASSO), support vector machine-recursive feature elimination (SVM-RFE), and random forests (RF). LASSO is a regression analysis algorithm that uses regularization to improve the accuracy of prediction and was performed using the “glmnet” package in R, in which the minimum absolute shrinkage was considered optimal (21). SVM is a supervised machine learning technique that is widely used for regression and classification, and the RFE algorithm is often applied to avoid overfitting. SVM-RFE was performed through the “e1071” package in R, with fivefold cross-validation (22). RF is an ensemble learning method that is based on the construction of many classification trees and was performed using the “randomForest” package in R, with genes awarded importance scores >2.0 being screened out (23). Finally, the genes identified using all three classification models were selected as potential gene biomarkers for further analysis.

### Diagnostic value of the candidate biomarkers for diabetic kidney disease

To test the predictive value of the identified gene biomarkers, we plotted receiver operating characteristic (ROC) curves for each using the mRNA expression data for 64 samples from patients with DKD and 44 samples from controls that comprised the training set. The GSE47185 and GSE30122 data set was then used as the validation set to test the efficacy of prediction using the identified gene biomarkers in more depth. The area under the ROC curve (AUC) was calculated to evaluate the diagnostic efficacy of the gene biomarkers identified using the algorithms. ROC curves were plotted using the “pROC” package in R, and a two-sided *P*-value of <0.05 was taken to indicate statistical significance.

### Renal immunohistochemical staining

To further understand the difference in expression of identified gene biomarkers in DKD and control kidney tissues, immunohistochemical (IHC) staining was conducted. The DKD kidney tissues were obtained from the patients with DKD diagnosed by renal biopsy pathology, while the normal kidney tissues were obtained from normal tissue next to kidney cancer. For IHC staining, all 50 DKD and 30 normal tissues from the kidney were fixed in 10% formalin for 24 hours and embedded in paraffin, then were cut into 5 μm thickness sliced sections. Paraffin-embedded tissue slides were deparaffinized in xylene and hydrated by increasing the alcohol concentrations. Tissue sections were treated with sodium citrate (pH = 6) and hydrogen peroxide for 15 min, then washed with PBS for antigen retrieval. Tissue sections were incubated in 5% BSA for 2 h (24). Subsequently, the tissue sections were treated with primary anti-DUSP1 and anti-PRKAR2B (1:200 dilution, the antibodies were purchased from Abcam) and incubated overnight at 4°C. Subsequently, the sections were incubated with horseradish peroxidase-conju-gated secondary antibodies for 60 min at room tempera- ture and stained through a horseradish peroxidase-DAB kit. All the stained sections were examined using an OLYMPUS cellSens Entry microscopy system. IHC staining was analyzed by Image-Pro Plus 6.0 software using a semiquantitative scoring method based on the staining intensity and the percentage of positively-stained cells (25).

### GSVA and GSEA analyses for the candidate biomarkers

To further illustrate the biological processes related to the candidate biomarkers, we divided the DKD samples into high and low expression groups based on the median values of the candidate biomarkers’ expressions respectively, and the GSVA and GSEA analyses were performed. The gene sets of “c5.go.bp.v7.4.symbols” were downloaded from the MSigDB database to enrich the biological process pathways (26). GSVA algorithm was used to analyze the data set to find biological process pathways with significant differences between samples (27).

### Immune cells infiltration

The CIBERSORT, XCELL, and TIMER algorithms were used to analyze the immunological characteristics of the DKD groups and control groups. CIBERSORT is the most widely used immune infiltration algorithm. It uses the linear support vector regression to deconvolute the tissue expression matrix and can accurately quantify the abundance scores of 22 types of immune cells for each sample (28). XCELL is a new method for cell-type enrichment analysis using single-sample gene set enrichment analysis. It could calculate the enrichment scores for 64 cell types, including not only 34 types of immune cells but also 30 stromal and other cells (29). Tumor immune estimation resource (TIMER) is a tool which integrates multiple state-of-theart algorithms for immune infiltration estimation (30). Subsequently, the “ggplot2” package in R was used for principal components analysis (PCA) clustering studies of the immune cell infiltration matrix. A heat map of the immune cell infiltration matrix by different methods was plotted using the “pheatmap” package to show the allocation of immune cell infiltrates in the respective kidney specimens. The correlations among the infiltrating immune cells identified were also plotted by the “pheatmap” package in the form of a heatmap. The “vioplot” package in R was used to draw a violin plot to aid visualization of the difference in immune cell infiltration between samples from patients with DKD and controls. Spearman’s rank correlation analysis in R was used to further analyze the relationships between the key diagnostic genes and immune infiltrating cells, and the “ggstatsplot” and “ggplot2” packages were used to aid visualization of the results.

## Results

### Identification of DEGs in samples from patients with diabetic kidney disease and controls

**FIGURE 1:**
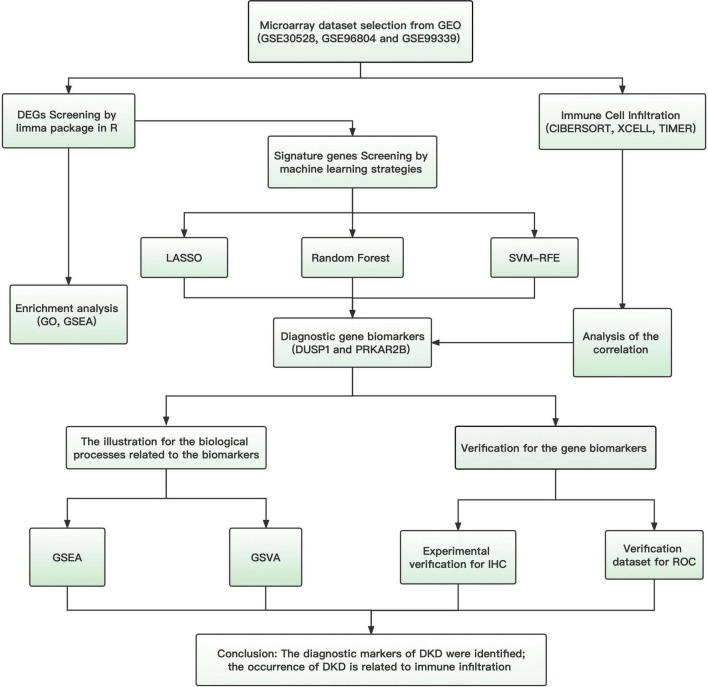
Study flow chart.

**FIGURE 2:**
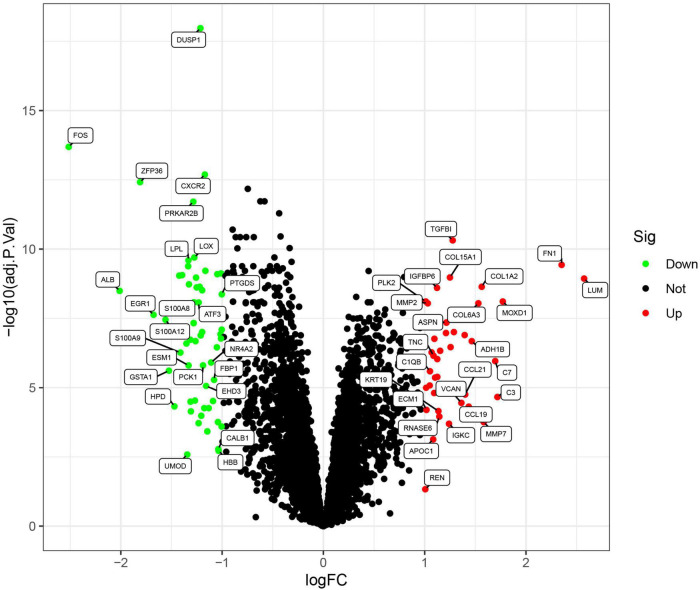
Volcano plots of the differentially expressed genes. Red: genes upregulated in diabetic kidney disease (DKD); green: genes downregulated in DKD.

The flowchart of this study was shown in Figure 1. Data from a total of 64 samples from patients with DKD and 44 controls obtained from three GEO data sets (GSE30528, GSE96804, and GSE99339) were retrospectively analyzed. The DEGs were identified using the limma package after the elimination of batch effects. A total of 95 DEGs were identified; 39 genes were significantly upregulated, and 56 were significantly downregulated (Figure 2).

### Functional correlation analysis

**FIGURE 3:**
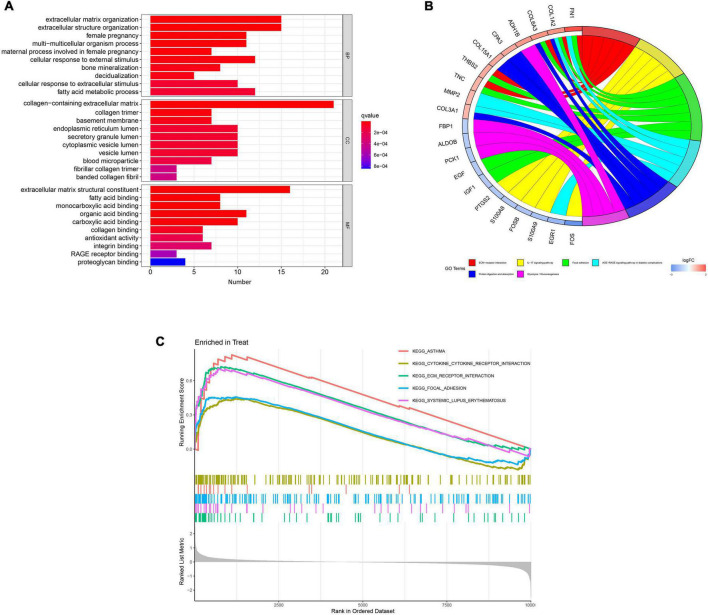
Results of functional enrichment analyses. **(A)** Gene Ontology enrichment analysis of the differentially expressed genes. **(B)** Kyoto Encyclopedia of Genes and Genomes pathway enrichment analysis results. **(C)** Gene set enrichment analysis (GSEA) profiles, showing the five significant GSEA sets.

Gene Ontology functional enrichment, KEGG pathway enrichment, and GSEA were used to investigate the mechanisms involved in the pathogenesis of DKD. The ten most significant biological process (BP), cellular component (CC), and molecular function (MF) terms in the GO functional enrichment for the DEGs were identified (Figure 3A). KEGG pathway enrichment revealed that the DEGs principally represented extracellular matrix (ECM)-receptor interaction, the interleukin (IL)-17 signaling pathway, focal adhesion, the advanced glycation end-product (AGE)-receptor for AGEs signaling pathway in diabetic complications, protein digestion and absorption, and glycolysis/gluconeogenesis (Figure 3B). Furthermore, GSEA identified the following principal enriched pathways: asthma, cytokine-cytokine receptor interaction, ECM receptor interaction, focal adhesion, and systemic lupus erythematosus (Figure 3C). All these findings suggest that the immune response plays a significant role in DKD.

### Identification and verification of diagnostic markers

**FIGURE 4:**
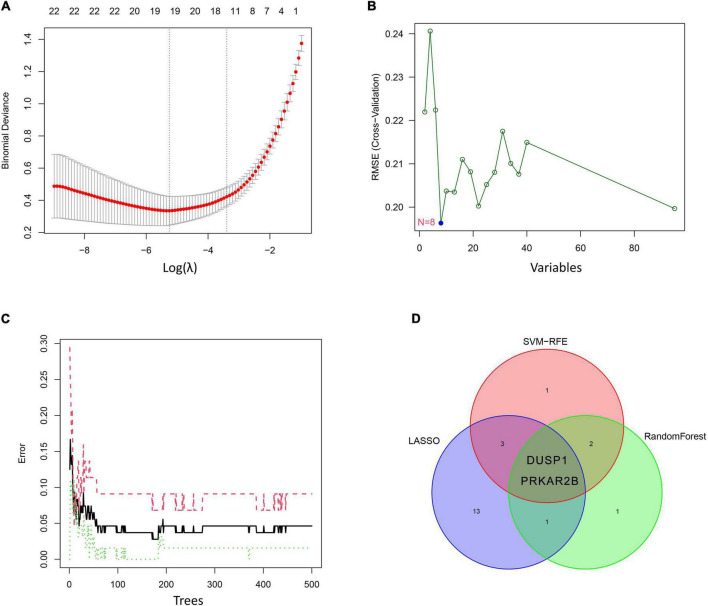
Candidate diagnostic marker genes identified using the three algorithms. **(A)** Least absolute shrinkage and selection operator (LASSO) logistic regression algorithm. **(B)** Support vector machine-recursive feature elimination (SVM-RFE) algorithm. **(C)** Random forest (RF) algorithm. **(D)** Venn diagram showing the overlaps in the candidate diagnostic genes identified using the three algorithms.

Three different algorithms were used to identify potential diagnostic markers for DKD. Nineteen genes among the DEGs were identified as potential biomarkers using the LASSO logistic regression algorithm (Figure 4A), eight genes were identified as potential biomarkers using the SVM-RFE algorithm (Figure 4B), and six genes were identified as potential biomarkers using the RF algorithm (Figure 4C). Two genes, *DUSP1* and *PRKAR2B*, were identified using all three algorithms and were therefore regarded as strong candidate predictors of DKD (Figure 4D).

**FIGURE 5:**
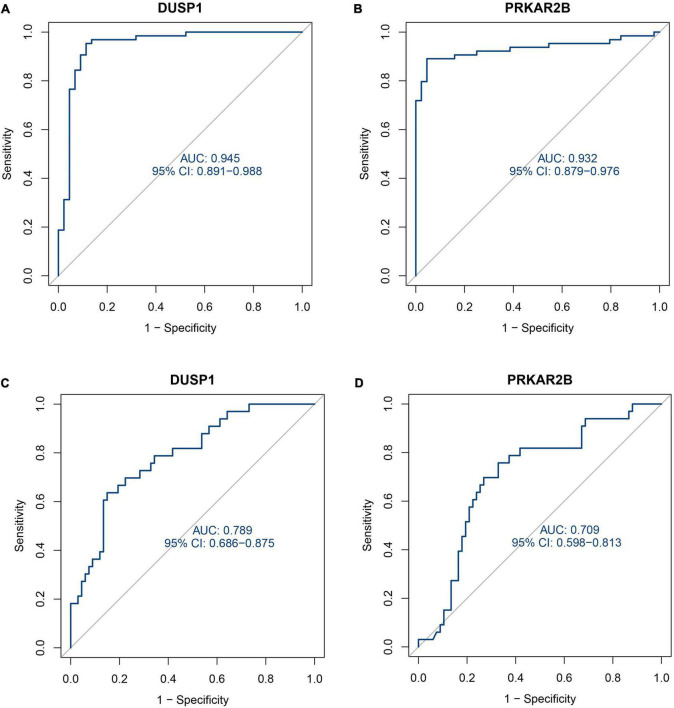
Receiver operating characteristic (ROC) curves describing the diagnostic efficacy of the two candidate diagnostic marker genes. **(A)** ROC curve for *DUSP1* using the training set. **(B)** ROC curve for *PRKAR2B* using the training set. **(C)** ROC curve for *DUSP1* using the validation set. **(D)** ROC curve for *PRKAR2B* using the validation set.

As shown in Figures 5A,B, the expression of these two genes was effective at discriminating DKD from control samples, with AUCs of 0.945 (95% CI 0.891–0.988) for *DUSP1* and 0.932 (95% CI 0.879–0.976) for *PRKAR2B*. To further evaluate the diagnostic efficacy of the two candidate biomarkers, the GSE47185 and GSE30122 datasets were merged as a validation set, which comprised 33 samples from patients with DKD and 67 samples from controls. As shown in Figures 5C,D, ROC curves for *DUSP1* and *PRKAR2B* were also plotted for the validation set and yielded AUCs of 0.789 (95% CI 0.686–0.875) and 0.709 (95% CI 0.598–0.813), respectively. These results indicate that expression of the two genes show good potential in the diagnosis of DKD.

### Renal immunohistochemical staining

**FIGURE 6:**
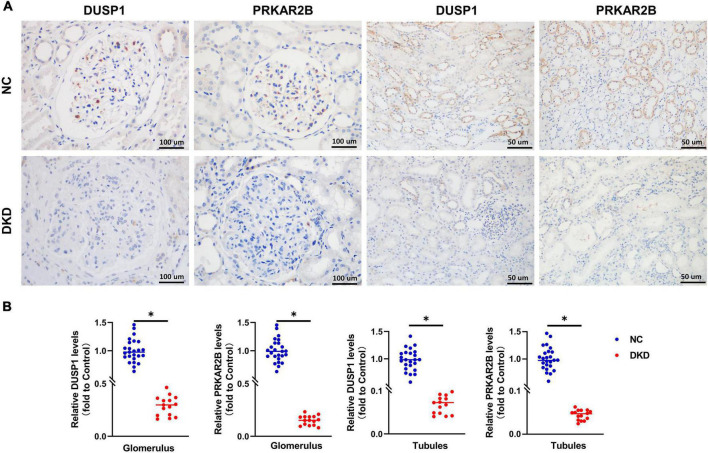
Clinical validation of the identified gene biomarkers expression in DKD kidney tissues and normal kidney tissues. **(A)** Representative images and **(B)** statistical analyses of immunohistochemical staining for DUSP1 and PRKAR2B. *P* < 0.05.

Immunohistochemical staining was performed in 50 DKD kidney tissues and 30 normal kidney tissues. Compared with the normal, patients with DKD presented with significantly lower expression levels of the DUSP1 and PRKAR2B (Figures 6A,B).

### GSVA and GSEA analyses for the candidate biomarkers

**FIGURE 7:**
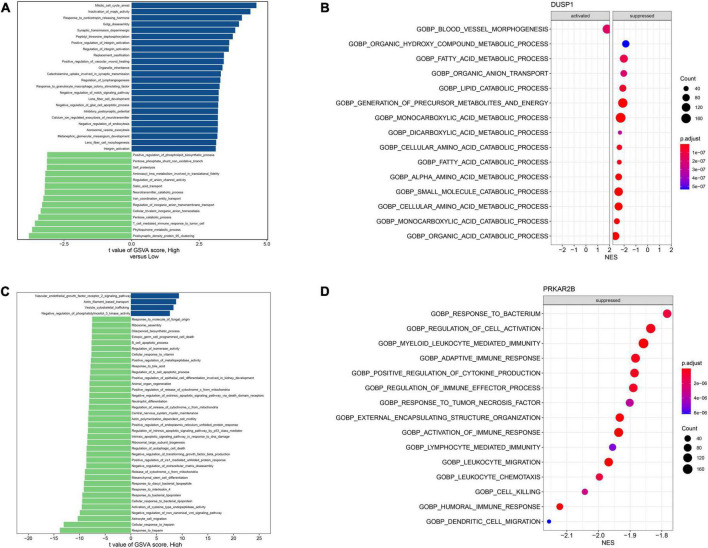
Exploring the biological processes related to the two candidate diagnostic marker genes. **(A)** GSVA analysis for the biological processes related to DUSP1. **(B)** GSEA analysis for the biological processes related to DUSP1. **(C)** GSVA analysis for the biological processes related to PRKAR2B. **(D)** GSEA analysis for the biological processes related to PRKAR2B.

The results of exploring the biological processes related to DUSP1 using GSVA and GSEA were shown in Figures 7A,B respectively, while the results of PRKAR2B were shown in Figures 7C,D respectively. The results showed that the expression of both DUSP1 and PRKAR2B was closely related to renal function, such as the regulation of glomerular filtration, glomerular epithelial cell development, renal system process involved in regulation of blood volume, etc.

### Analysis of immune cell infiltration

**FIGURE 8:**
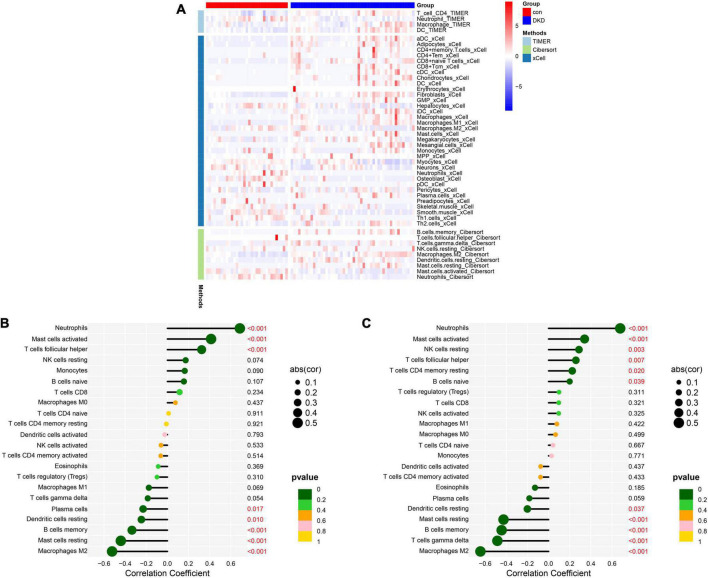
Analysis of immune cell infiltration. **(A)** The heatmap of immune cells with differential infiltration between DKD patients and controls based on CIBERSORT, XCELL and TIMER algorithms. **(B)** Correlations between *DUSP1* expression and the extent of infiltration of immune cell subtypes. **(C)** Correlations between *PRKAR2B* expression and the extent of infiltration with immune cell subtypes.

The heatmap of immune cells with differential infiltration between DKD patients and controls based on CIBERSORT, XCELL and TIMER algorithms is shown in Figure 8A. In CIBERSORT algorithm, the correlation heatmap for the 22 immune cell subtypes demonstrated that two immune cell subtypes (activated mast cells and resting mast cells) were negatively correlated (Supplementary Figure 1), and the violin plot of the differentially infiltrated immune cells demonstrated that B memory cells, gamma delta T cells, resting natural killer (NK) cells, macrophages, and resting mast cells were present in larger numbers in the DKD samples than in the control samples, whereas follicular helper T cells, resting dendritic cells, activated mast cells, and neutrophils were present in smaller numbers (Supplementary Figure 2). The results of XCELL and TIMER algorithms also support the finding of CIBERSORT that macrophages infiltrate more in DKD samples and neutrophils infiltrate more in controls. Violin plots of the different infiltrated immune cells identified by XCELL and TIMER algorithms were shown in Supplementary Figures 3, 4 respectively.

The relationships between the candidate diagnostic marker genes and the infiltrating immune cells were also analyzed. DUSP1 expression positively correlated with neutrophil infiltration (*r* = 0.69, *P* < 0.001), activated mast cell infiltration (*r* = 0.41, *P* < 0.001), and T follicular helper cell infiltration (*r* = 0.32, *P* < 0.001) and negatively correlated with plasma cell infiltration (*r* = –0.23, *P* = 0.017), resting dendritic cell infiltration (*r* = −0.25, *P* = 0.010), memory B cell infiltration (*r* = –0.34, *P* < 0.001), resting mast cell infiltration (*r* = –0.44, *P* < 0.001), and M2 macrophage infiltration (*r* = −0.52, *P* < 0.001) (Figure 8B). PRKAR2B expression positively correlated with neutrophil infiltration (*r* = 0.68, *P* < 0.001), active mast cell infiltration (*r* = 0.34, *P* < 0.001), resting NK cell infiltration (*r* = 0.29, *P* = 0.003), T follicular helper cell infiltration (*r* = 0.26, *P* = 0.007), resting memory CD4 + T cell infiltration (*r* = 0.22, *P* = 0.020), and naïve B cell infiltration (*r* = 0.20, *P* = 0.039) and negatively correlated with resting dendritic cell infiltration (*r* = –0.20, *P* = 0.037), resting mast cell infiltration (*r* = –0.43, *P* < 0.001), memory B cell infiltration (*r* = –0.44, *P* < 0.001), gamma delta T cell infiltration (*r* = –0.48, *P* < 0.001), and M2 macrophage infiltration (*r* = –0.65, *P* < 0.001) (Figure 8C).

## Discussion

Diabetic kidney disease is the most common cause of CKD and ESRD worldwide (31) and involves the hyperplasia or hypertrophy of various cell types in the glomerulus and tubules, thickening of the glomerular and tubular basement membranes, and expansion of the tubulointerstitial and mesangial compartments (32). Because the early clinical manifestations of DKD are easy to ignore, it has usually progressed to an advanced stage by the time it is recognized, which is associated with a poor prognosis. In addition, immune cell infiltration plays a significant role in the development of DKD (33). Therefore, the prognosis of patients with DKD could be improved by the identification and use of specific diagnostic markers and a fuller characterization of the pattern of immune cell infiltration into the glomerular region of the kidneys of patients with DKD. Recently, bioinformatics and CIBERSORT tools have been increasingly used to identify novel diagnostic markers and to analyze the patterns of immune cell infiltration in tissues (34, 35). However, few studies have focused on characterizing the relationships between candidate gene biomarkers and the immune cell infiltration in DKD (36). Therefore, we aimed to identify candidate diagnostic biomarkers for DKD and further investigate the role of immune cell infiltration in DKD.

We downloaded expression profile datasets from the GEO database and identified a total of 95 DEGs. GSEA showed that the following principal pathways were enriched: asthma, cytokine-cytokine receptor interaction, ECM receptor interaction, focal adhesion, and systemic lupus erythematosus. Cui et al. (37) showed that the DEGs in glomerular and tubular samples from patients with DKD were primarily associated with pathways involved in ECM-receptor interactions and cytokine-cytokine receptor interactions, which is consistent with the present findings and suggests that these pathways may play significant roles in the progression of DKD.

Least absolute shrinkage and selection operator logistic regression is a machine learning method that determines the variable by finding λ when the classification error is the smallest (38). SVM is a machine learning method of pattern recognition and estimation of function that operates within the framework of statistical learning theory and structural risk minimization (39). RF is an algorithm that uses an ensemble decision tree, in which random subsets are drawn from the data, with replacement (40). These three algorithms are principally used to identify the most specific DEGs in order to facilitate construction of a superior diagnostic model. In the present study, we identified *DUSP1* and *PRKAR2B* as candidate diagnostic marker genes using the above three algorithms, and to further evaluate their diagnostic efficacy, we used a validation set. The results of this analysis suggested that our integration strategy was reliable.

The dual-specificity phosphatase (DUSP) family is the largest group of protein phosphatases that specifically regulate mitogen-activated protein kinase (MAPK) activity in human cells (41). One study of the mechanism of fibrosis in DKD showed that DUSP1 plays an anti-fibrotic role in the HK-2 proximal tubular cell line by inactivating both the p38MAPK and extracellular signal-related protein kinase 1/2 pathways (42). Angiotensin II-stimulated proteasome activity results in DUSP1 degradation and the subsequent activation of signal transducer and activator of transcription 1 in T cells, leading to the induction of T helper 1 differentiation (43). Moreover, the inhibition of DUSP1 expression or function in T cells may be beneficial for the treatment of T cell-mediated autoimmune diseases such as multiple sclerosis (44). Thus, DUSP1 influences the activity of immune cells and the process of fibrosis in tissues by regulating MAPK, and it has therefore become a potential therapeutic target in some immune diseases. Therefore, DUSP1 may well influence the pathogenesis of DKD.

cAMP-dependent protein kinase type II-beta (PRKAR2B) is a member of the protein kinase A (PKA) family that is primarily expressed in the brain and adipose tissue (45) and phosphorylates Ser/Thr residues of target proteins (46). In a previous study, it was shown that a hypoxia-induced reduction in *PRKAR2B* transcription is sufficient to increase PKA activity *via* hypoxia-inducible factor (HIF)-1α, without affecting intracellular cAMP concentration (47). In another study, it was shown that PRKAR2B increases the expression of HIF-1α, which provides a growth advantage in prostate cancer by increasing the Warburg effect (48). In DKD, ischemia and inflammation in the glomeruli and vascular lesions reduce the oxygen supply, resulting in an increase in HIF-1α expression, which assists cells to cope with hypoxia. However, hyperglycemia can reduce the stability of HIF-1α, which leads to tissue fibrosis (49). Therefore, we speculate that PRKAR2B may be involved in the progression of DKD under hypoxic conditions. The results of previous studies have suggested that DUSP1 and PRKAR2B play significant roles in the activities of immune cells and in the progression of disease in tissues (50–52). Therefore, these two genes may represent useful diagnostic markers of DKD, but further clinical studies are needed to confirm the efficacy of their use for diagnostic purposes.

The results of immune cell infiltration found that there was greater infiltration with B memory cells, gamma delta T cells, resting NK cells, macrophages, and resting mast cells, and less infiltration with T follicular helper cells, resting dendritic cells, activated mast cells, and neutrophils, which may be involved in the development and progression of DKD. Current studies generally demonstrated that the mechanism of neutrophils in regulation the pathogenesis of DKD is mediated by inflammatory response. A study including 22 renal biopsies of DKD proved that the infiltration of neutrophils increased in interstitial, peritubular, and capillary regions (53). Neutrophil extracellular traps (NETs) are the bactericidal mechanism after neutrophil death. A previous study showed that neutrophils isolated from diabetic humans and mice were primed to produce NETs (54). The increase of Biomarkers of NETs correlates with DKD severity, which NETs promote NLRP3 inflammasome activation and glomerular endothelial dysfunction under high glucose stress *in vitro* and *in vivo* (14). Additionally, neutrophils may also migrate into the kidneys of patients with DKD because their spontaneous adhesion increases (55), which is followed by abnormal activation and the secretion of proinflammatory cytokines, degranulation, and the release of reactive oxygen species (56). To sum up, neutrophils may involve in the occurrence of DKD. In our study, we found that the proportion of neutrophils was relatively lower in DKD samples, which is consistent with the results of Wang et al. on immune infiltration in DKD (57). We suggested that it may be a potential limitation of the current immune infiltration algorithms, because the higher proportion of macrophages in DKD patients makes the proportion of other immune cells, including neutrophils, appear lower (57). The mechanism of other immune cells in regulation the pathogenesis of DKD have also been showed in previous studies. One previous animal study showed that CD4 + CD8 + T cells and dendritic cells are present in the glomeruli of diabetic mice, along with immunoglobulin (Ig)G and IgG + B cells, which may suggest that the IgG present in the glomeruli may be produced by the infiltrating B cells *in situ* (58). Some features of DKD, such as the high expression of intracellular adhesion molecule 1 and monocyte chemoattractant protein 1 by renal tubular cells, may increase the recruitment of macrophages (59, 60), which then respond to the locally high concentrations of glucose, AGEs, and oxidized low-density lipoprotein by secreting proinflammatory cytokines (61, 62). Eller et al. (63) demonstrated that the depletion of regulatory T cells using an anticluster of differentiation 25 monoclonal antibody accelerates the progression of renal injury, involving increases in glomerular hyperfiltration and albuminuria. One study of streptozotocin-induced diabetes in mice showed that T helper 17 cells may protect diabetic kidneys by reducing and modifying the inflammatory response (64). Mast cells have also been shown to be present in large numbers in the renal interstitium of patients with DKD, and their number has been shown to correlate with serum creatinine concentration (65). The degranulation of mast cells has been demonstrated in renal biopsies from patients with type 2 diabetes and various stages of nephropathy (66).

An analysis of the correlations between the expression of the two candidate diagnostic marker genes and the extent of infiltration with subtypes of immune cells revealed close negative correlations of *DUSP1* and *PRKAR2B* expression with the extent of M2 macrophages infiltration and positive correlations with neutrophil infiltration. Studies on renal biopsies showed that M1 and M2 macrophages infiltrating in the renal tissue of DKD were increased in a process-dependent manner, and the activation status of M1 and M2 macrophages was positively correlated with the progression of DKD (67). Another previous vitro cell experiment proved that M2 macrophage could improve high glucose-induced podocytes injury via secreting exosomal miR-25-3p to activate autophagy of the cells through suppressing DUSP1 expression (68). The above indicates that with the progression of DKD, M2 macrophages act as a protective factor to inhibit the expression of DUSP1, thereby ameliorating the renal damage of DKD. Unfortunately, few study revealed the interaction between PRKAR2B and M2 macrophages in DKD. Previous studies have shown that neutrophils and mast cells play significant roles in the progression of DKD (55, 56, 66). Macrophages and certain types of T cells may be increasingly recruited during the progression of DKD (58–60). Therefore, we infer that *DUSP1* and *PRKAR2B* may be involved in regulating the infiltration of immune cells in the kidney, and thus influence the development and progression of DKD. However, this hypothesis requires further testing in preclinical and clinical contexts.

We have used novel bioinformatic methods to filter potential diagnostic marker genes and validated them through IHC, and also investigated the relationship between the identified markers and immune cell infiltration. Nevertheless, there were a number of limitations to the present study. First, clinical information regarding the samples and the contributing patients was not available for the datasets obtained from the GEO database; therefore, the influences of other disease factors on the expression of genes could not be evaluated. Second, our study performed IHC in DKD patients and controls but did not detect the expression of these two biomarkers in the blood samples, the early detection potential of DUSP1 and PRKAR2B of DKD needs to be further explored. Third, the public database we used did not provide follow up information for us to explore relationship of these biomarkers with disease prognosis. Therefore, further prospective clinical studies on the prognostic relevance of DUSP1 and PRKAR2B to DKD need to be conducted in the future.

In conclusion, we have shown that DUSP1 and PRKAR2B may represent potential diagnostic markers of DKD. We have also shown that immune cells such as B memory cells, macrophages, resting mast cells, follicular helper T cells, activated mast cells, and neutrophils may be involved in the development and progression of DKD. Furthermore, the expression of DUSP1 and PRKAR2B was found to significantly correlate with the numbers of immune cells such as neutrophils and M2 macrophages in the kidneys of such patients. DUSP1 and PRKAR2B may be involved in regulating the infiltration of immune cells in the kidney, and thus influence the development and progression of DKD.

## Data availability statement

The datasets presented in this study can be found in online repositories. The names of the repository/repositories and accession number(s) can be found in the article/Supplementary material.

## Ethics statement

The studies involving human participants were reviewed and approved by Ethical Institutions of the First Hospital of Jilin University (Changchun, China; approval no. 2022−417). Written informed consent for participation was not required for this study in accordance with the national legislation and the institutional requirements.

## Author contributions

SF and YC conceived, designed the research, conducted the bioinformatics analysis and wrote original draft of the manuscript. XW, JH, and JY curated and analyzed the data. SS and HW helped in data collection and manuscript editing. ZX supervised the study and reviewed the manuscript. All authors contributed to the study and approved the final version.

## Footnotes

1
http://www.ncbi.nlm.nih.gov/geo/
