# Supplementary material for: Identification of diagnostic gene biomarkers and immune infiltration in patients with diabetic kidney disease using machine learning strategies and bioinformatic analysis
Source: Front Med (Lausanne). 2022 Sep 29;9:918657. doi: 10.3389/fmed.2022.918657 (PMC9556813; doi:10.3389/fmed.2022.918657)
Supplement: Supplementary file 1 [file Data_Sheet_1.docx]

Supplementary Material

## Supplementary Figures
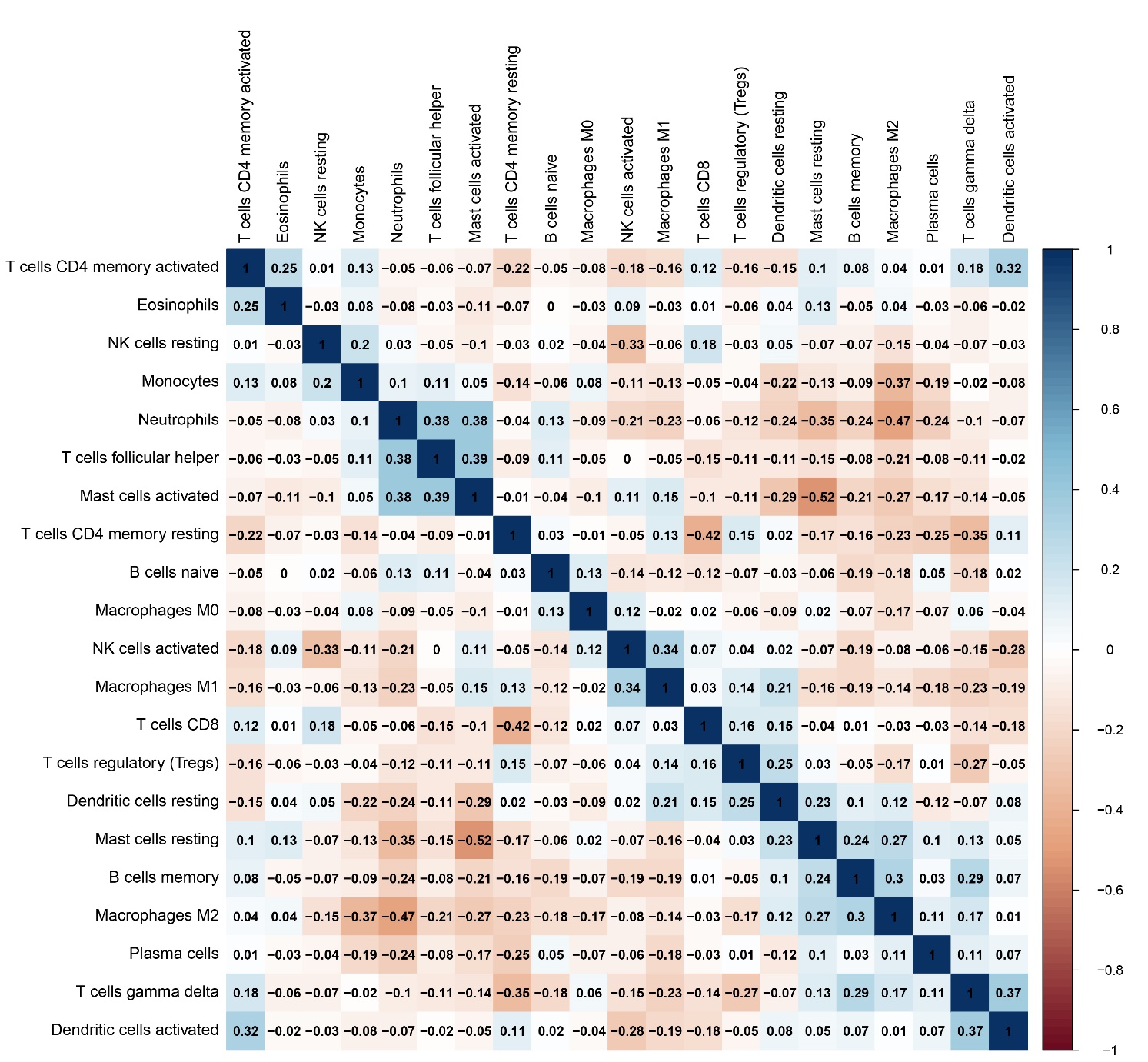


## Supplementary Figure 1. Correlation heatmap for the infiltration by all the subtypes of immune cells. Blue squares represent positive correlations, and red squares represent negative correlations; the deeper the color is, the stronger the correlation.


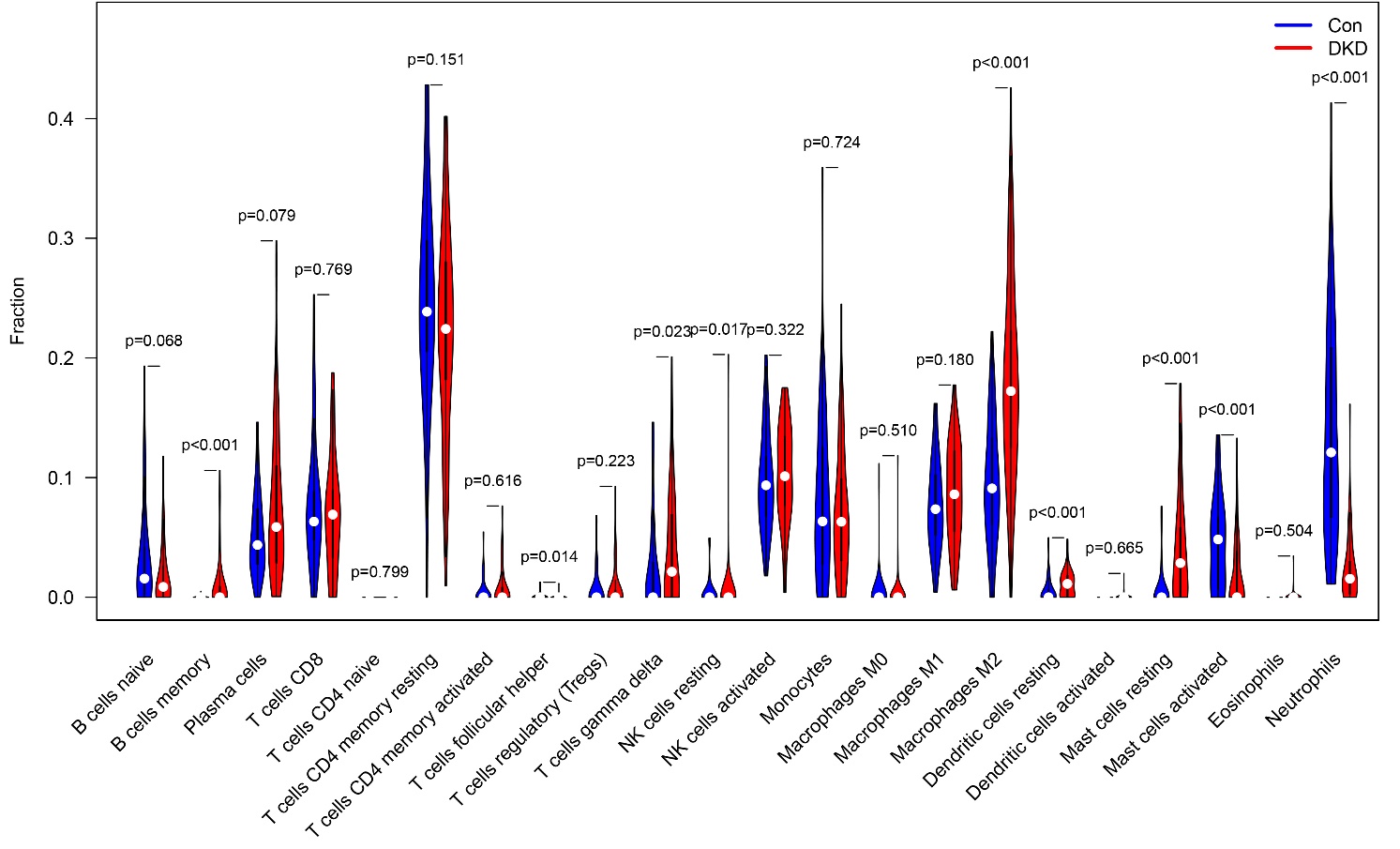


**Supplementary Figure 2.** Violin plots of the different infiltrated immune cells identified by CIBERSORT algorithms. *P*-values < 0.05 are indicative of significant differences in the level of infiltration of an immune cell subtype between the two groups of samples.


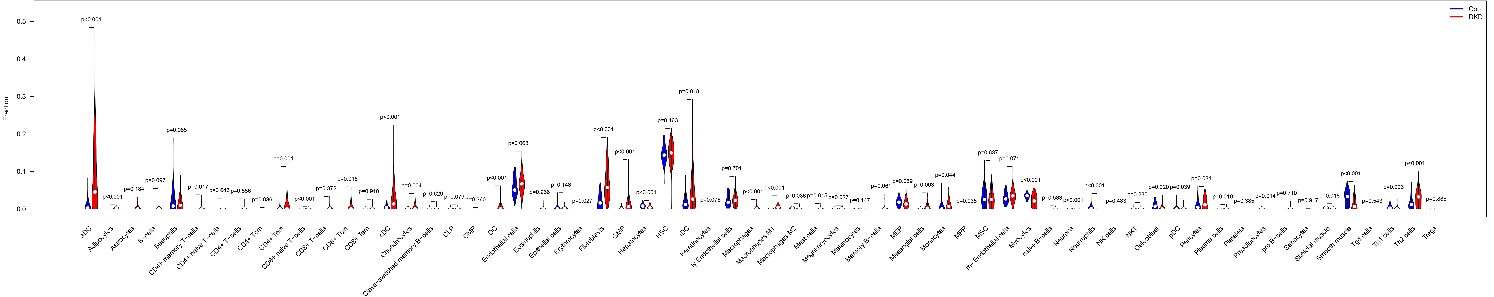


**Supplementary Figure 3.** Violin plots of the different infiltrated immune cells identified by XCELL algorithms. *P*-values < 0.05 are indicative of significant differences in the level of infiltration of an immune cell subtype between the two groups of samples.


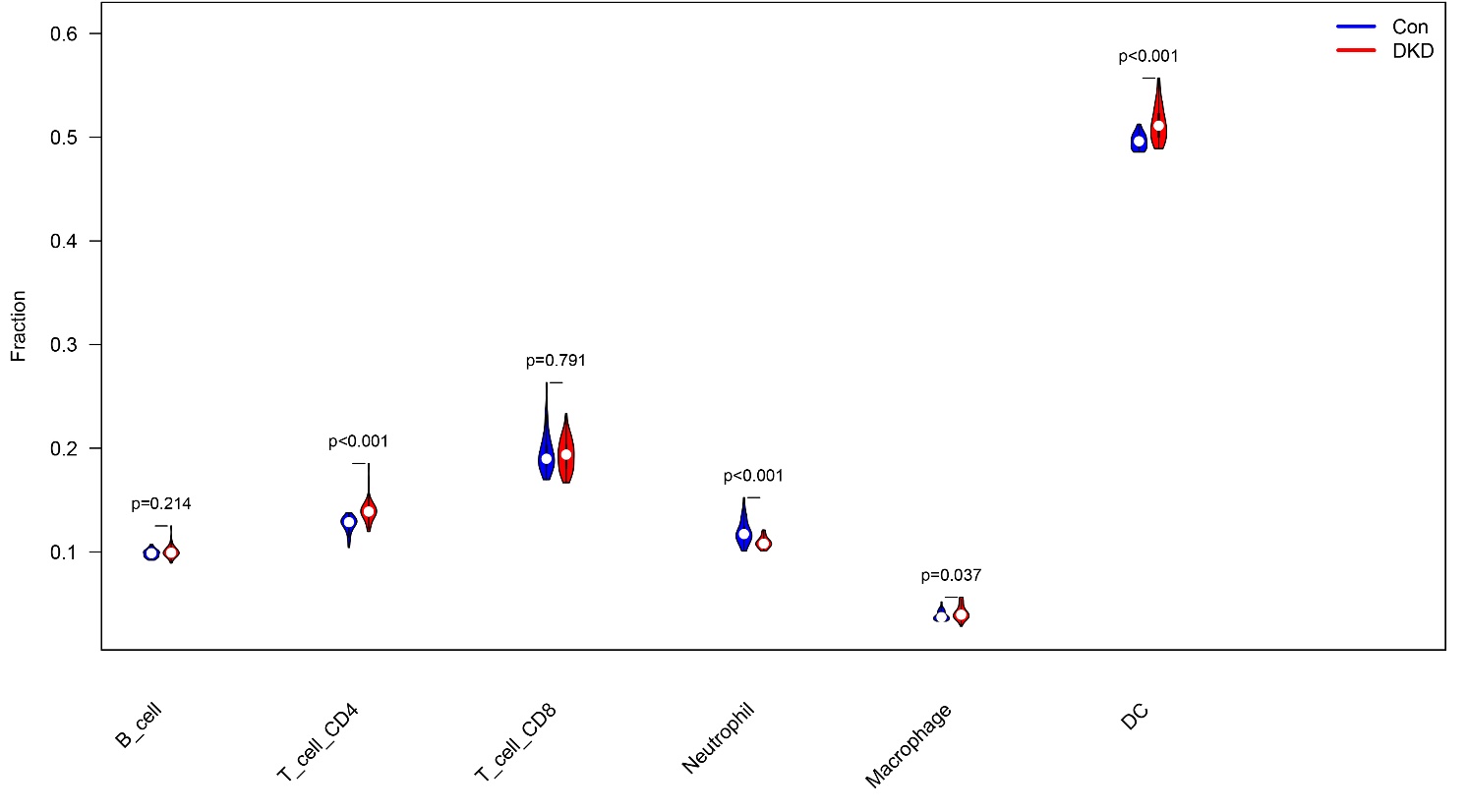


**Supplementary Figure 4.** Violin plots of the different infiltrated immune cells identified by TIMER algorithms. *P*-values < 0.05 are indicative of significant differences in the level of infiltration of an immune cell subtype between the two groups of samples.
